# Medium-/Long-Term Effects of a Specific Exercise Protocol Combined with Patient Education on Spine Mobility, Chronic Fatigue, Pain, Aerobic Fitness and Level of Disability in Fibromyalgia

**DOI:** 10.1155/2014/474029

**Published:** 2014-01-29

**Authors:** Erika Giannotti, Konstantinos Koutsikos, Maurizia Pigatto, Maria Elisa Rampudda, Andrea Doria, Stefano Masiero

**Affiliations:** ^1^Rehabilitation Unit, Department of Neurosciences, University of Padua, Via Giustiniani 3, 35128 Padua, Italy; ^2^Department of Rheumatology, University of Padua, Via Giustiniani 2, 35128 Padua, Italy; ^3^Department of Rehabilitation Medicine, University of Padua, Via Giustiniani 2, 35131 Padua, Italy

## Abstract

*Objective*. To propose a rehabilitation protocol able to produce immediate and long-term beneficial effects on level of disability and overall performance in ADLs. *Materials and Methods*. Forty-one FM patients were randomized to an exercise and educational-behavioral programme group (experimental group, EG = 21) or to a control group (CG = 20). Each subject was evaluated before, at the end (T1), and after 6 months (T6) from the conclusion of the rehabilitation treatment using the Fibromyalgia Impact Questionnaire (FIQ), the visual analogue scale (VAS), the Health Assessment Questionnaire (HAQ), the fatigue severity scale (FSS), the 6-minute walking test (6MWT), tender points count (TPC), and spinal active range of motion. The exercise protocol included 20 sessions consisting in self-awareness, stretching, strengthening, spine flexibility, and aerobic exercises, which patients were subsequently educated to perform at home. *Results*. The two groups were comparable at baseline. At T1, the EG showed a positive trend in FIQ, VAS, HAQ, and FSS scales and significant improvement in 6MWT and in most spinal active range of motion measurements (*P* between 0.001 and 0.04). The positive results were maintained at the follow-up. *Conclusion*. The proposed programme was well tolerated and produced immediate and medium-term beneficial effects improving function and strain endurance. This trial is registered with DRKS00005071 on DRKS.

## 1. Introduction 

Fibromyalgia (FM) is a chronic widespread pain disorder, commonly associated with comorbid symptoms, including fatigue, nonrestorative sleep, poor balance, cognitive/memory problems, psychological distress, and impaired physical function [1], along with a reduced quality of life [2, 3]. Given the complex symptom presentation and the multiple comorbidities associated, Häuser et al. [4] recommended a multidisciplinary team in the FM treatment.

Current guidelines for FM treatment management follow core principles of comprehensive assessment, education, goal setting, multimodal management including pharmacological (e.g., pregabalin, duloxetine, and milnacipran) and nonpharmacological therapies (e.g., physical activity, behavioral therapy, sleep hygiene, and education), regular education, and monitoring of treatment response [5, 6].

Physical exercise is one of the most widely recognized and beneficial forms of nonpharmacological therapy [7–9], effective in reducing pain and depression and producing positive effects on physical function, fitness, and global health [10], particularly in patients affected by rheumatic disease [11].

The most consistent results have been demonstrated for aerobic and strengthening exercise that, when combined with stretching, had equivalent effects on limiting pain severity among patients with FM [12, 13].

Moreover the combination of aerobic exercise, strengthening, and flexibility has also been shown to improve psychological health status, preventing depression, and health-related quality of life [14, 15].

Despite the strong empirical evidence for exercise benefits, the optimal exercise program for patients with FM is yet to be determined [16]. While planning a specific rehabilitation treatment, individual characteristics such as physical fitness, function and symptom severity, and goals should be taken into consideration in order to gain optimal benefits, ensure long-term effects and adherence, and support in adopting active lifestyles that include regular exercise [10, 17].

Few studies have evaluated the long-term effects of rehabilitation, as authors are often challenged by patients' low compliance and important adverse effects, such as postexercise pain, leading to high dropout rates [18–20].

We hypothesized that a group exercise program, physiotherapist supervised, characterized by different types of exercise (e.g., aerobic and stretching) in the same session and a gradual progression from low-intensity exercise, using the “start low and go slow” approach, as suggested by Jones and Liptan [21], associated with telephone calls and home exercise diaries, might help to motivate FM patients to continue at home the exercise programme learned, thus promoting a long-term adherence to the rehabilitation treatment.

The study's primary outcome was to evaluate the efficacy of a specific rehabilitation protocol, based on an association of aerobic workout, muscle strengthening and flexibility exercises, combined with patient education, in producing immediate and long-term beneficial effects on level of disability, spine mobility, endurance, and overall performance in activities of daily living (ADLs).

Secondary outcomes included evaluating the possible adverse effects and the adherence at the proposed rehabilitation treatment.

## 2. Materials and Methods

This is a randomized case-control study, approved by our Hospital Ethics Committee conformed to the principles of the Declaration of Helsinki and informed consent of all patients was obtained.

### 2.1. Subjects

FM subjects recruited had a confirmed diagnosis of FM, based on the American College of Rheumatology (ACR) 2010 criteria [1, 22].

The following inclusion criteria for patient selection were used: patients with the diagnosis of FM, aged between 35 and 65 yrs and with body mass index (BMI) between 18 and 35 kg/m^2^.

Exclusion criteria included (a) diabetes; (b) other rheumatic diseases including severe osteoarthritis (altering the ambulation pattern) and severe osteoporosis (T score > 3); (c) severe musculoskeletal alterations (determining skeletal deformities); (d) users of assistive devices to perform daily activities; (e) orthopaedic surgery, such as spine or hip/knee surgery in the previous year; and (f) patients who had attended physical therapy and rehabilitation treatments or had modified their usual FM pharmacological therapy in the previous 3 months of the enrollment.

The 41 patients meeting the inclusion criteria were allocated randomly to an experimental group (EG, *n* = 21) that received our rehabilitation protocol, combining physical exercise and an educational-behavioral programme, and a control group (CG, *n* = 20) that did not receive the above treatment. Randomization was based on a computer-generated random number table.

Each subject was evaluated before the rehabilitation training (baseline, T0), at the end (T1), and 6 months after the conclusion of the rehabilitation protocol (T6) by the same rheumatologist (tender point count (TPC), disability, pain, sleep disorders, weariness, and stiffness) and physiatrist (body composition, spine mobility, and functional capacity).

During this period (from T0 to T6) patients in the CG refrained from conducting rehabilitation treatments or continuous physical activity while EG was encouraged to perform at home the exercise programme learned (from T1 to T6) at least three times a week.

### 2.2. Clinical Evaluation

#### 2.2.1. Body Composition

Weight and height have been measured; body mass index (weight (kg)/height (m^2^)) have been calculated.

#### 2.2.2. Tenderness, Pain, Sleep Disorders, Weariness, and Stiffness

Tenderness was assessed by applying about 4 kg finger pressure at 18 tender points until the fingernail bed blanched. The *TPC*, total count of positive tender points, was then recorded for each participant [3].

The* visual analogue scale *(VAS) is a simple assessment tool consisting of a 10 cm line with 0 on one end, representing no symptom, and 10 on the other, representing the highest intensity ever experienced, which a patient marks to indicate the severity of a specific manifestation; this scale has been used to evaluate pain (in the last 24 hours), sleep disorders, weariness, and stiffness in the last week.

#### 2.2.3. Disability

The* Fibromyalgia Impact Questionnaire *(FIQ) is a validated, disease-specific and self-administered questionnaire, comprising 10 subscales of disabilities and symptoms. FIQ was used for evaluation of range of symptoms experienced by FM patients and responses to therapy and includes 20 questions that assess functionality with ADLs, work difficulty, general feelings of well-being, sleep quality, and the severity of symptoms including pain, fatigue, depression, anxiety, and stiffness [23].

In this work we used the Italian version of the FIQ. The scores of each item are standardized on a scale ranging from 0 to 10 with higher scores indicating a higher level of impairment [24].

The *fibromyalgia assessment status* (FAS) is a composite disease-specific and simple self-administered index that combines a patient's assessment of fatigue, sleep disturbances, and pain. It is considered a valid and reliable measure for assessing treatment effects in patients with FM that can be used to obtain reliable information concerning the course of the disease [25].

*Fatigue severity scale* (FSS) is a measurement of fatigue impact on functioning. It was developed by Krupp et al. [26] and is a short nine-item self-report questionnaire with simple and quick application [27].

#### 2.2.4. Functional Capacity

Functional capacity was measured through the *Health Assessment Questionnaire *(HAQ) and the *6-minute walk test* (6MWT).

HAQ is a self-report functional status measure and the domain of disability is assessed by the eight categories of dressing, arising, eating, walking, hygiene, reach, grip, and common activities.

For each of these categories, patients report the amount of difficulty (0–3 with higher scores indicating severe disability) they have in performing two or three specific activities [28].

The 6MWT is a simple, safe, and low cost test that has already been used in previous studies of patients with FM and has shown to have good reliability [29, 30], in particular the distance walked, during the 6MWT, has been suggested to reflect the ability to perform activities of daily living (ADLs) because, in general, ADLs are performed at submaximal levels [31]. For the present study, the 6MWT was performed in a plane corridor of 30 meters in length, following the recommendations of the American Thoracic Society [32].

#### 2.2.5. Spine Mobility

Thoracic kyphosis (TK), lumbar lordosis (LL), and active spinal range of motion (ROM), in particular flexion-extension, right and left inclination, and right and left rotation, were evaluated by means of a validated pocket compass needle goniometer (IncliMed, Patent no. 0001331516, University of Padua) [33, 34].

### 2.3. Rehabilitation Programme

The EG group performed a specific exercise protocol combined with patient education 2 days/week (60 min per session) for a 10-week period (Table 1). Every session of the rehabilitation programme was conducted in group and supervised by a physiotherapist.

The programme (20 sessions) was divided in three parts: the first part (1–7 sessions) was focused on patient education, where the physiotherapist explained the characteristics of FM including information on the symptoms, diagnosis, and treatment, physical and mental health, and instructed patients how to perform the various proposed exercises. In the second (8–14 sessions) and third (15–20 sessions) part, the physiotherapist dedicated the first 10 minutes to test the correct execution of exercises learned and to ask if there were pain during or after the home exercises and if some exercises were not tolerated.

The rehabilitation programme was characterized by a gradual introduction of novel exercises with a progressive increase in intensity (from low to moderate intensity reached in the last six sessions of the intervention). Starting from session 8 strengthening exercises were intensified and from session 15 aerobic exercises were added. Exercise included stretching, strengthening, active and passive mobilization, spine flexibility and aerobic training, applied on the upper body, trunk, and lower body, for improving cardiovascular endurance, muscle strength and stretch, and joint range of motion (Table 1).

### 2.4. Participant Retention and Adherence

To maximize adherence, several strategies were implemented, including telephone calls following missed sessions, use of home exercise diaries, and the control of patients' pain rate before and after each session. Moreover, in the diaries patients of both groups had to keep track of the use of analgesic/anti-inflammatory drugs only when they had taken any, in particular, for EG both during the rehabilitation treatment (before or after each session) and during the exercise at home (in the period T1–T6).

### 2.5. Statistical Analysis

Data were organized with a Spreadsheet (OpenOffice Calc) and analyzed with R 2.14.0. The significance level was set at *P* ≤ 0.05. At first step common statistical indexes (average, median, and standard deviation) were calculated then comparisons were carried out with ANOVA or *t*-test in conjunction with Shapiro-Wilk test to asses normality of data and Levene test to asses homogeneity of variances. Considering each variable as dependent variable, we mainly applied two factors ANOVA, with a factor “within” (TVal) and a factor “between” (Group), using the R package [35]. For post hoc analysis we firstly performed a graphical analysis by interaction plots; then we used the pairwise *t*-test for multiple comparisons with Holm's correction or one-way ANOVA on subgroup defined by each factor levels combination. When data did not meet the ANOVA requirements we used nonparametric methods: Friedman's test followed by Wilcoxon's test for paired-data or Mann-Whitney *U* test.

## 3. Results

### 3.1. Baseline

**Figure 1:**
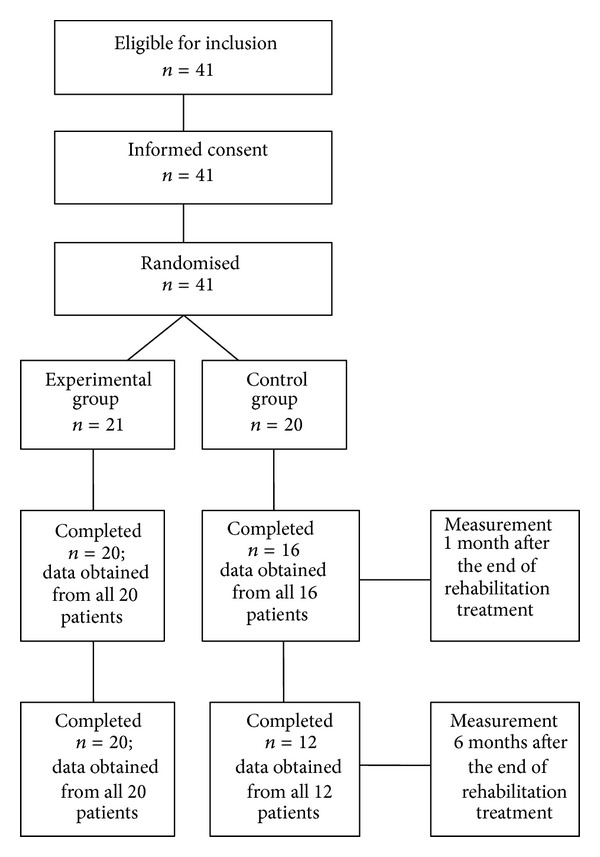
Study flowchart.

Of the 41 participants enrolled in this study, 20 patients (19 F, 1 M; mean age 52.8 years ± 10.69; mean BMI 24.4 ± 3.46) in the EG and 12 patients in the CG (11 F, 1 M; mean age 51.3 years ± 6.3; mean BMI 23.4 ± 4.24) completed the trial (Figure 1). The two groups were comparable at baseline with regard to their sociodemographic variables and primary outcomes (Table 2). The reasons for the 9 missing were the following In the EG, 1 patient was missed for undergoing surgery. In the CG, 1 patient for pregnancy, 1 for lost contacts due to residence variation, and 6 for personal problems and lack of sustained motivation to complete the trial.

### 3.2. Posttreatment Results

No adverse effects of exercise, such as an increase in symptoms (e.g., pain, stiffness, and fatigue) and musculoskeletal problems (e.g., plantar fasciitis and impingement syndrome) were observed during the treatment and follow-up periods.

EG group reported to have performed at home the exercise programme learned, with variable frequency (with a frequency of at least 3 times a week).

In the EG we observed a general positive trend in almost every outcome parameter considered (Tables 3 and 4). In particular we found a statistically significant decrease in the *TPC* and *sleep disorders* in the EG between T0 and T1 (*P* = 0.034 and *P* = 0.007, resp.), while between T0 and T6 the difference was very close to significance. Concerning *stiffness*, a significant effect of the treatment was found in the form of a VAS score reduction between T0 and T1 in EG (*P* = 0.013) compared to CG and between T0 and T6 in EG (*P* = 0.022).

On the other hand the EG showed a VAS score reduction for pain and weariness, although not statistically significant. Moreover, based on the observation of patients' diaries and historic information acquired, we noted that 90% of subjects in the baseline groups reported an assumption of NSAIDs at least once a day in the month prior to the rehabilitation start. During the following months, in the EG we noted a gradual reduction in percentage. In particular, at T1, 75% of patients continued with a daily NSAINDs consumption, while in T6 the value was further reduced to 70%. In the CG the percentage did not vary significantly, reported at 83% of daily NSAIDs consumption.

With regard to the disability parameters, the *FSS* score showed a gradual decrease in T1 and T6, the *FIQ* score reduction was very close to significance between T0 and T1 (*P* = 0.056) while the *FAS* scale presented statistically significant effects, in terms of reduced score, between T0 and T1 (*P* = 0.026), and these values were maintained in T6.

In terms of functional capacity, we observed a score reduction in the HAQ score for the EG, both in T1 and in T6 compared to T0 and a statistically significant increase in gait speed in T1 and T6 compared to T0 in the EG (*P* < 0.001). On the contrary, the CG maintained stable values in HAQ scale and decreased gait speed at T6 follow-up.

Regarding the *spine mobility*, the EG showed an increase in all assessed ROM parameters, with a statistical significance in extension, lateral inclination, and rotation values both in T1 and T6 (*P* between 0.003 and 0.045).

## 4. Discussion

In patients affected by FM, exercise brings beneficial effects on pain, physical function, and fatigue [36] and a multicomponent therapy, combining psychological therapy with rehabilitation programme, is strongly recommended [37].

Although several authors report the efficacy of rehabilitation programmes [38, 39], poor compliance and high dropout rates are evident in many exercise studies. For the above reasons in the literature there are very few data putting in evidence the medium-/long-term effect of physical therapy [13, 18].

In the present study, we analyzed the immediate and medium-/long-term effects of an experimental rehabilitation treatment characterized by educational-behavioral indications and a tailored exercise routine concentrated in relatively short sessions. Based on the hypothesis that our programme could be significantly improved by encouraging and promoting increased interaction between participants, exercises were conducted in group. Our aim was also to assess whether such an approach could stimulate patient's compliance, thus rendering it easily reproducible in regular basis, even at home.

Compared to previous studies afflicted by a high number of dropouts and poor compliance due to increased pain after exercise in FM patients [40–42], we observed the absence of significant adverse effects (like pain and stiffness exacerbation) which led to an excellent adherence of the EG to the proposed treatment and during the entire observation period. Furthermore, we observed that the EG reported a reduction of the monthly consumption of NSAID/analgesic drugs from T1 to T6, passing from a 90% at baseline to 70% at the 6-month follow-up. This fact, highlighted by the low dropout rate in EG (1 patient) as opposed to the CG (8 patients), might indicate that our specific combined rehabilitation treatment was indeed very well tolerated and accepted by the participants.

Moreover, the strategies implemented, including telephone calls following missed sessions and use of home exercise diaries, apparently acted in synergy with the educational-behavioral programme in maximizing patients' compliance. Participants affirmed that they continued exercise at home and applied the indications given during each session in most of the ADL.

In agreement with Pankoff et al. [43] we also came across an increase in covered distance during the 6MWT parallel to a FIQ scale score reduction in the EG, which reflects the higher ability of FM patients to execute ADLs [24, 32].

The positive variation of speed after the rehabilitation treatment is associated with reduced stiffness and increased mobility of the spine, despite the fact that HAQ and FSS scores have not shown a statistically significant reduction. The improvement of endurance during gait, noticed at the 6MWT, is probably incidental to the increase of spine ROM and the decrease of stiffness. On the other hand, we hypothesize that the discrepancy between the evaluation scales, FSS and HAQ (subjective questionnaires) and 6MWT (objective performance test), is mainly determined by the fact that the two different types of measurement are not easily comparable.

Data from several studies suggest that FM pain is primarily maintained by a dysregulated central nervous system (CNS) [44, 45]. McLoughlin et al. [46] provided preliminary data supporting a positive relationship between physical activity and CNS pain processing and suggesting that physically active FM patients appear to maintain their ability to modulate pain while those who are less active do not. To date, the few available lines of evidence in the literature render the above conclusions speculative.

In the present study the EG showed a significant reduction of TPC, and pain score, assessed with VAS scale, decreased after the rehabilitation programme (T1), results that appear in agreement with a recent paper [47] in which authors highlighted that physical activity was positively related to brain activity during pain modulation. In contrast with previous studies [48], none of the EG patients reported exacerbation of pain or physical exertion during the 6MWT.

It is interesting observing that most of the positive effects of the proposed rehabilitation treatment, in particular on spinal active ROM, stiffness, and gait speed, were well maintained at the 6-month follow-up, suggesting that patients continued to benefit from it, not just as a direct result of the rehabilitation programme but also thanks to the continuation of physical activity at home. Moreover we established that the behavioral-educational intervention, leading to better self-awareness and acquisition of healthy habits even at home, has had a positive impact on pain, muscle stiffness, fatigue, sleep disorders, and overall physical function, thus reducing disability.

We feel, though, that a few considerations should be kept in mind when interpreting our results. In fact, our study presents some obvious limits such as the low population number, especially for the CG, in which we have had a high dropout rate, the brief follow-up period (6 months), and the lack of a direct comparison between our tailored intervention and a standard physiotherapy programme [49]. Therefore, the above limitations may represent a good start point for future research, which should be oriented to establish the longer-term efficacy of this specific programme, comparing its effects versus other nontailored treatments and to implement a more thorough analysis of its effective possibility to reduce analgesic drugs consumption.

## 5. Conclusion

The proposed rehabilitation treatment has been demonstrated to be safe and well tolerated, with a good adherence in time. Given the good results obtained, moreover maintained at the 6 month follow-up, our programme has been proven effective and could be proposed as an adjunctive treatment in the multidisciplinary management of patients affected by FM syndrome.

## Figures and Tables

**Table 1:** Rehabilitation programme.

Sessions 1–7	Sessions 8–14	Sessions 15–20
Patient's education (self-awareness) (10 min)	Patient's education (10 min)	Patient's education (10 min)

Information about exercises: illustrated description by the physiotherapist about exercises to be learned and performed at home	Verifying the correct execution of exercises learned, presence of pain during or after the home exercises, and overall exercise tolerance.	Verifying the correct execution of exercises learned, presence of pain during or after the home exercises, and overall exercise tolerance.

Warm-up (15 min)	Warm-up (10 min)	Warm-up (10 min)

(1) Pulmonary exercises, cardiorespiratory fitness:	(1) Pulmonary exercises, cardiorespiratory fitness:	(1) Pulmonary exercises, cardiorespiratory fitness:
(a) inspiration through the nose and expiration through the mouth, (b) normal expiration through the nose and normal expiration through the mouth, (c) respiration through the chest and abdomen, and (d) deep breathing and then expiration through the mouth slowly deep breathing.	(a) inspiration through the nose and expiration through the mouth, (b) normal expiration through the nose and normal expiration through the mouth, (c) respiration through the chest and abdomen, and (d) deep breathing and then expiration through the mouth slowly deep breathing.	(a) inspiration through the nose and expiration through the mouth, (b) normal expiration through the nose and normal expiration through the mouth, (c) respiration through the chest and abdomen, and (d) deep breathing and then expiration through the mouth slowly deep breathing.
(2) Chest and shoulders stretching exercises	(2) Chest and shoulders stretching exercises	(2) Chest and shoulders stretching exercises
(2 series of 10 repetitions).	(2 series of 10 repetitions).	(2 series of 10 repetitions).
(3) Spine and upper and lower limbs stretching exercises	(3) Spine and upper and lower limbs stretching exercises	(3) Spine and upper and lower limbs stretching exercises
(2 series of 10 repetitions)	(2 series of 10 repetitions)	(2 series of 10 repetitions)

Main period (25 min)	Main period (30 min)	Main period (30 min)

(1) Exercises to mobilize the back and limbs—crucial for maintaining joint range of movement (2 series of 10 repetitions):	(1) Exercises to mobilize the back and limbs—crucial for maintaining joint range of movement (2 series of 10 repetitions):	(1) Exercises to mobilize the back and limbs—crucial for maintaining joint range of movement (2 series of 10 repetitions):
*Cervical area and thoracolumbar area:* lateral flexion and rotation, flexion-extension. *Shoulder and upper limbs*: ab/adduction, flexion, elevation, and circumduction. *Coxofemoral, knee, and ankle:* *add/abduction, rotation, and flexo-extension. *	*Cervical side and thoraco-lumbar area:* lateral flexion and rotation, flexion-extension. *Shoulder and upper limbs*: ab/adduction, flexion, elevation, and circumduction. *Coxofemoral, knee, and ankle:* *add/abduction, rotation, and flexo-extension. *	*Cervical side and thoraco-lumbar area:* lateral flexion and rotation, flexion-extension. *Shoulder and upper limbs*: ab/adduction, flexion, elevation, and circumduction. *Coxofemoral, knee, and ankle:* *add/abduction, rotation, and flexo-extension. *
(2) Stretching exercises for the anterior and posterior pelvic girdle muscle chain and muscles of the lower limbs.	(2) Stretching exercises for the anterior and posterior pelvic girdle muscle chain and muscles of the lower limbs. Muscle strengthening for spine and lower limbs	(2) Stretching exercises for the anterior and posterior pelvic girdle muscle chain and muscles of the lower limbs. Muscle strengthening for spine and lower limbs
(2 repetitions for 50/60 seconds)	(2 repetitions for 50/60 seconds)	(2 repetitions for 50/60 seconds)
		Aerobic exercises (exercise bike): 10 min at 70% of max fc

Cooling down (10 min)	Cooling down (10 min)	Cooling down (10 min)

Respiratory exercises; spine and limb stretching exercises	Respiratory exercises; spine and limb stretching exercises	Respiratory exercises; spine and limb stretching exercises

**Table 2:** Demographic characteristics of the two groups.

Characteristics	Experimental group (*n* = 20)	Control group (*n* = 12)
Age, mean ± SD, years	52.8 ± 10.6	51.3 ± 6.3
Female/male	19/1	11/1
Duration of FM related symptoms before diagnosis, mean ± SD, years	7.6 ± 8.8	7.1 ± 5.2
Married/cohabiting, %	90.2	92.3
BMI %	24.3	23.4
Employed, % yes/no	65.1/34.9	67.4/32.6

BMI: body mass index; FM: fibromyalgia; SD: standard deviation.

**Table 3:** Physiatrical evaluation results.

Measures	Groups	T0 (mean ± SD)	T1 (mean ± SD)	T6 (mean ± SD)	*P * (T0-T1)	*P * (T0–T6)
ROM (degrees)						
Flexion	EG	37.75 ± 14.30	43.95 ± 11.26	45.40 ± 9.53	ns	ns
	CG	35.33 ± 15.69	34.00 ± 10.61	32.67 ± 11.77	ns	ns
Extension	EG	3.75 ± 6.07	7.40 ± 6.49	8.45 ± 6.02	**0.040 **	**0.008**
	CG	4.75 ± 5.38	4.33 ± 4.25	4.33 ± 2.67	ns	ns
Inclination L	EG	22.00 ± 7.31	27.60 ± 6.97	28.80 ± 8.48	**0.014**	**0.003**
	CG	22.42 ± 8.39	22.42 ± 4.85	22.42 ± 4.70	ns	ns
Inclination R	EG	20.50 ± 6.01	26.60 ± 7.35	26.25 ± 6.87	**0.025**	**0.031**
	CG	21.08 ± 8.33	22.17 ± 7.55	22.33 ± 8.90	ns	ns
Rotation L	EG	39.75 ± 12.93	47.00 ± 8.11	52.75 ± 12.91	**0.045**	**0.004**
	CG	39.83 ± 12.81	39.83 ± 9.68	40.75 ± 9.81	ns	ns
Rotation R	EG	40.30 ± 11.90	47.25 ± 11.22	49.15 ± 9.72	ns	**0.041**
	CG	41.25 ± 14.25	39.08 ± 10.45	42.33 ± 9.85	ns	ns
6MWT (speed: m/s)	EG	1.05 ± 0.19	1.30 ± 0.24	1.24 ± 0.22	**<0.001**	**<0.001**
	CG	1.12 ± 0.24	1.13 ± 0.24	1.03 ± 0.21	ns	ns
HAQ (0–60)	EG	13.95 ± 8.79	10.40 ± 8.72	10.15 ± 10.32	ns	ns
	CG	12.50 ± 6.79	12.75 ± 8.49	11.75 ± 6.96	ns	ns
FSS (9–63)	EG	52.05 ± 11.44	47.90 ± 10.62	47.25 ± 11.29	ns	ns
	CG	56.00 ± 7.03	55.50 ± 7.40	54.58 ± 9.25	ns	ns

SD: standard deviation; ROM: range of motion; EG: experimental group; CG: control group; L: left; R: right; 6MWT: 6-minute walking test; HAQ: Health Assessment Questionnaire; FSS: fatigue severity scale; ns: nonsignificant.

In bold: statistically significant values.

**Table 4:** Rheumatological evaluation results.

	Groups	T0 (mean ± SD)	T1 (mean ± SD)	T6 (mean ± SD)	*P * (T0-T1)	*P * (T0–T6)
Pain (VAS, 0–10)	EG	6.10 ± 2.07	5.25 ± 2.47	5.80 ± 1.99	ns	ns
	CG	6.08 ± 1.62	5.50 ± 2.43	5.42 ± 2.87	ns	ns
Sleep disorders (VAS, 0–10)	EG	6.8 ± 2.65	4.6 ± 3.14	6.3 ± 2.99	**0.007**	0.056
	CG	6.92 ± 3.53	5.00 ± 3.05	6.08 ± 3.42	ns	ns
Stiffness (VAS, 0–10)	EG	7.50 ± 1.7	6.40 ± 2.56	6.85 ± 1.57	**0.013**	**0.022**
	CG	8.00 ± 1.21	6.67 ± 0.89	6.17 ± 2.59	ns	ns
Weariness (VAS, 0–10)	EG	7.70 ± 1.87	6.15 ± 2.58	7.05 ± 1.93	ns	ns
	CG	7.58 ± 2.75	7.58 ± 2.23	7.08 ± 1.88	ns	ns
FIQ (0–100)	EG	62.66 ± 14.42	55.45 ± 12.20	48.75 ± 17.43	0.056	ns
	CG	59.09 ± 15.63	50.92 ± 19.96	56.94 ± 14.47	ns	ns
FAS (0–10)	EG	6.61 ± 1.61	5.25 ± 1.86	6.17 ± 1.67	**0.026**	ns
	CG	6.42 ± 2.08	5.98 ± 1.55	5.73 ± 2.23	ns	ns
TPC (0–18)	EG	12.70 ± 4.65	9.35 ± 5.02	9.55 ± 5.45	**0.034**	0.054
	CG	13.67 ± 4.50	11.83 ± 5.77	12.17 ± 5.77	ns	ns

SD: standard deviation; EG: experimental group; CG: control group; VAS: visual analogue scale; FIQ: Fibromyalgia Impact Questionnaire; FAS: Fibromyalgia Assessment Status; TPC: tender point count; ns: nonsignificant.

In bold: statistically significant values.

## References

[1] Wolfe F, Clauw DJ, Fitzcharles M-A (2010). The American College of Rheumatology preliminary diagnostic criteria for fibromyalgia and measurement of symptom severity. *Arthritis Care and Research*.

[2] Mease PJ, Arnold LM, Crofford LJ (2008). Identifying the clinical domains of fibromyalgia: contributions from clinician and patient delphi exercises. *Arthritis Care and Research*.

[3] Wolfe F, Smythe HA, Yunus MB (1990). The American College of Rheumatology 1990. Criteria for the classification of fibromyalgia. Report of the Multicenter Criteria Committee. *Arthritis and Rheumatism*.

[4] Häuser W, Bernardy K, Arnold B, Offenbächer M, Schiltenwolf M (2009). Efficacy of multicomponent treatment in fibromyalgia syndrome: a meta-analysis of randomized controlled clinical trials. *Arthritis Care and Research*.

[5] Arnold LM, Clauw DJ, Dunegan LJ, Turk DC (2012). A framework for fibromyalgia management for primary care providers. *Mayo Clinic Proceedings*.

[6] Culpepper L (2012). Management of fibromyalgia in primary care. *Journal of Clinical Psychiatry*.

[7] Sarzi-Puttini P, Atzeni F, Salaffi F, Cazzola M, Benucci M, Mease PJ (2011). Multidisciplinary approach to fibromyalgia: what is the teaching?. *Best Practice and Research: Clinical Rheumatology*.

[8] Robinson RL, Kroenke K, Mease P (2012). Burden of illness and treatment patterns for patients with fibromyalgia. *Pain Medicine*.

[9] Cazzola M, Atzeni F, Salaffi F, Stisi S, Cassisi G, Sarzi-Puttini P (2010). What kind of exercise is best in fibromyalgia therapeutic programmes? A practical review. *Clinical and Experimental Rheumatology*.

[10] Busch AJ, Webber SC, Brachaniec M (2011). Exercise therapy for fibromyalgia. *Current Pain and Headache Reports*.

[11] Masiero S, Boniolo A, Wassermann L, Machiedo H, Volante D, Punzi L (2007). Effects of an educational-behavioral joint protection program on people with moderate to severe rheumatoid arthritis: a randomized controlled trial. *Clinical Rheumatology*.

[12] Hooten WM, Qu W, Townsend CO, Judd JW (2012). Effects of strength vs aerobic exercise on pain severity in adults with fibromyalgia: a randomized equivalence trial. *Pain*.

[13] Busch AJ, Overend TJ, Schachter CL (2009). Fibromyalgia treatment: the role of exercise and physical activity. *International Journal of Clinical Rheumatology*.

[14] Häuser W, Thieme K, Turk DC (2010). Guidelines on the management of fibromyalgia syndrome—a systematic review. *European Journal of Pain*.

[15] Sañudo B, Galiano D, Carrasco L, de Hoyo M, McVeigh JG (2011). Effects of a prolonged exercise programe on key health outcomes in women with fibromyalgia: a randomized controlled trial. *Journal of Rehabilitation Medicine*.

[16] Dagfinrud H, Kvien TK, Hagen KB (2005). The cochrane review of physiotherapy interventions for ankylosing spondylitis. *Journal of Rheumatology*.

[17] Mannerkorpi K (2009). Physical activity and body functions in patients with fibromyalgia syndrome. *Journal of Musculoskeletal Pain*.

[18] Häuser W, Klose P, Langhorst J (2010). Efficacy of different types of aerobic exercise in fibromyalgia syndrome: a systematic review and meta-analysis of randomised controlled trials. *Arthritis Research & Therapy*.

[19] Friedberg F, Williams DA, Collinge W (2012). Lifestyle-oriented non-pharmacological treatments for fibromyalgia: a clinical overview and applications with home-based technologies. *Journal of Pain Research*.

[20] Bircan Ç, Karasel SA, Akgün B, El Ö, Alper S (2008). Effects of muscle strengthening versus aerobic exercise program in fibromyalgia. *Rheumatology International*.

[21] Jones KD, Liptan GL (2009). Exercise interventions in fibromyalgia: clinical applications from the evidence. *Rheumatic Disease Clinics of North America*.

[22] Wolfe F, Häuser W (2011). Fibromyalgia diagnosis and diagnostic criteria. *Annals of Medicine*.

[23] Bennett R (2005). The Fibromyalgia Impact Questionnaire (FIQ): a review of its development, current version, operating characteristics and uses. *Clinical and Experimental Rheumatology*.

[24] Sarzi-Puttini P, Atzeni F, Fiorini T (2003). Validation of an Italian version of the fibromyalgia impact questionnaire (FIQ-I). *Clinical and Experimental Rheumatology*.

[25] Salaffi F, Sarzi-Puttini P, Girolimetti R, Gasparini S, Atzeni F, Grassi W (2009). Development and validation of the self-administered Fibromyalgia Assessment Status: a disease-specific composite measure for evaluating treatment effect. *Arthritis Research & Therapy*.

[26] Krupp LB, LaRocca NG, Muir-Nash J, Steinberg AD (1989). The fatigue severity scale. Application to patients with multiple sclerosis and systemic lupus erythematosus. *Archives of Neurology*.

[27] Dittner AJ, Wessely SC, Brown RG (2004). The assessment of fatigue: a practical guide for clinicians and researchers. *Journal of Psychosomatic Research*.

[28] Fries JF, Spitz P, Kraines RG, Holman HR (1980). Measurement of patient outcome in arthritis. *Arthritis and Rheumatism*.

[29] Enrichi PL, Sherrill DL (1998). Reference equations for the six-minute walk in healthy adults. *American Journal of Respiratory and Critical Care Medicine*.

[30] Mannerkorpi K, Svantesson U, Broberg C (2006). Relationships between performance-based tests and patients’ ratings of activity limitations, self-efficacy, and pain in fibromyalgia. *Archives of Physical Medicine and Rehabilitation*.

[31] Du H, Newton PJ, Salamonson Y, Carrieri-Kohlman VL, Davidson PM (2009). A review of the six-minute walk test: its implication as a self-administered assessment tool. *European Journal of Cardiovascular Nursing*.

[32] American Thoracic Society (2002). ATS statement: guidelines for the six-minute walk test. *American Journal of Respiratory and Critical Care Medicine*.

[33] Masiero S, Bonaldo L, Pigatto M, Lo Nigro A, Ramonda R, Punzi L (2011). Rehabilitation treatment in patients with ankylosing spondylitis stabilized with tumor necrosis factor inhibitor therapy. A randomized controlled trial. *Journal of Rheumatology*.

[34] Gravina AR, Ferraro C, Frizziero A, Ferraro M, Masiero S (2012). Goniometer evaluation of thoracic kyphosis and lumbar lordosis in subjects during growth age: a validity study. *Studies in Health Technology and Informatics*.

[35] Lawrence MA Easy analysis and visualization of factorial experiments. http://cran.r-project.org/web/packages/ez/index.html.

[36] Winkelmann A, Häuser W, Friedel E (2012). Physiotherapy and physical therapies for fibromyalgia syndrome. Systematic review, meta-analysis and guideline. *Schmerz*.

[37] Arnold B, Häuser W, Arnold M (2012). Multicomponent therapy of fibromyalgia syndrome. Systematic review, meta-analysis and guideline. *Schmerz*.

[38] Brosseau L, Wells GA, Tugwell P (2008). Ottawa panel evidence-based clinical practice guidelines for aerobic fitness exercises in the management of fibromyalgia: part 1. *Physical Therapy*.

[39] Busch AJ, Schachter CL, Overend TJ, Peloso PM, Barber KAR (2008). Exercise for fibromyalgia: a systematic review. *Journal of Rheumatology*.

[40] Richards S, Cleare A (2000). Treating fibromyalgia. *Rheumatology*.

[41] Nørregaard J, Lykkegaard JJ, Mehlsen J, Danneskiold-Samsøe B (1997). Exercise training in treatment of fibromyalgia. *Journal of Musculoskeletal Pain*.

[42] Wigers SH, Stiles TC, Vogel PA (1996). Effects of aerobic exercise versus stress management treatment in fibromyalgia: a 4.5 year prospective study. *Scandinavian Journal of Rheumatology*.

[43] Pankoff B, Overend T, Lucy D, White K (2000). Validity and responsiveness of the 6 minute walk test for people with fibromyalgia. *Journal of Rheumatology*.

[44] Cook DB, Lange G, Ciccone DS, Liu W-C, Steffener J, Natelson BH (2004). Functional imaging of pain in patients with primary fibromyalgia. *Journal of Rheumatology*.

[45] Petersel DL, Dror V, Cheung R (2011). Central amplification and fibromyalgia: disorder of pain processing. *Journal of Neuroscience Research*.

[46] McLoughlin MJ, Stegner AJ, Cook DB (2011). The relationship between physical activity and brain responses to pain in fibromyalgia. *Journal of Pain*.

[47] Ellingson LD, Shields MR, Stegner AJ, Cook DB (2012). Physical activity, sustained sedentary behavior, and pain modulation in women with fibromyalgia. *Journal of Pain*.

[48] Homann D, Stefanello JMF, Góes SM, Leite N (2011). Impaired functional capacity and exacerbation of pain and exertion during the 6-minute walk test in women with fibromyalgia. *Revista Brasileira de Fisioterapia*.

[49] van Koulil S, Effting M, Kraaimaat FW (2007). Cognitive-behavioural therapies and exercise programmes for patients with fibromyalgia: state of the art and future directions. *Annals of the Rheumatic Diseases*.

